# *Arthrographis* Infections in Humans—A Narrative Review

**DOI:** 10.3390/pathogens15010112

**Published:** 2026-01-20

**Authors:** Afroditi Ziogou, Alexios Giannakodimos, Ilias Giannakodimos, Andreas G. Tsantes, Stella Baliou, Petros Ioannou, Georgia Vrioni, George Samonis

**Affiliations:** 1 Department of Internal Medicine, 417 Army Equity Fund Hospital, 115 21 Athens, Greece; 2 Department of Cardiology, Tzaneio General Hospital of Piraeus, 185 37 Piraeus, Greece; 3 Department of Urology, Attikon General Hospital of Athens, 124 62 Athens, Greece; 4 Microbiology Department, “Saint Savvas” Oncology Hospital, 115 22 Athens, Greece; andreas.tsantes@yahoo.com; 5 School of Medicine, University of Crete, 710 03 Heraklion, Greece; 6 Department of Microbiology, Medical School, National and Kapodistrian University of Athens, 115 27 Athens, Greece; gvrioni@med.uoa.gr; 7 First Oncology Department, Metropolitan Hospital, Neon Faliron, 185 47 Athens, Greece

**Keywords:** *Arthrographis*, infection, meningitis, bloodstream, peritonitis, endocarditis, pneumonia, osteomyelitis

## Abstract

Background: *Arthrographis* spp. are environmental fungi commonly found in soil and compost. Infections caused by *Arthrographis* species remain an uncommon clinical occurrence. Although these infections are infrequent in the general population, their incidence appears to be elevated among immunocompromised patients or those with significant comorbidities. Objectives: This review seeks to examine all documented human cases of *Arthrographis* spp. infections, with particular focus on aspects such as epidemiology, microbiological features, resistance patterns, therapeutic approaches and associated mortality rates. Methods: A narrative review was performed based on data obtained from the PubMed/MedLine and Scopus databases. Results: A total of 21 articles reported *Arthrographis* spp. infections in 21 patients. The mean age of affected individuals was 43.62 years, with 66.6% being male. A history of trauma was the most common predisposing factor, present in 33.33% of cases. Fever and abscess formation were the predominant clinical manifestations (28.6%), followed by organ dysfunction in 19% of patients. In vitro, the yeast generally showed susceptibility to voriconazole and itraconazole, with a low rate of resistance to amphotericin B. Clinically, amphotericin B was the most frequently administered antifungal (55%), followed by voriconazole (40%) and itraconazole (30%). The overall mortality rate was 19%, while deaths directly attributable to the infection accounted for 14.3%. Conclusions: Due to the capacity of *Arthrographis* spp. to cause serious infections, it is important for healthcare providers to consider this organism when dimorphic yeast appears in biological specimens’ cultures, especially in patients with immunosuppression or significant underlying conditions, to facilitate timely and accurate diagnosis.

## 1. Introduction

*Arthrographis* species are environmental fungi commonly found in soil and compost, with *A. kalrae*, formerly known as *Oidiodendron kalrai*, being the most frequently encountered [1]. *A. kalrae* is a filamentous, asexual and slow-growing fungus that produces arthroconidia and blastoconidia [2,3]. Initially described in 1939 from a nail infection, it is generally an opportunistic pathogen that thrives in collagen-rich tissues like nails and corneas; however, it can also cause invasive infections such as pneumonia, endocarditis, meningitis and sinusitis. In recent years, an increase in invasive infections caused by this pathogen has been observed [4]. Immunosuppression increases the risk of infection, although healthy individuals can also be affected. Infections by *Arthrographis* species typically manifest as local lesions, but may also progress to systemic disease, especially in immunocompromised patients. Microbial differentiation is challenging due to its dimorphic nature [2]. Due to limited published literature and sparse antifungal susceptibility data, there is currently no consensus on the optimal management of *A. kalrae* infections; therefore, management is generally empirical, depending on the results of antifungal testing [5]. These infections carry a significant risk of mortality, particularly in patients with severe comorbidities and compromised immune status.

The main objective of this research is to compile and analyze all documented instances of human infections attributed to *Arthrographis* species, emphasizing trends in epidemiology and mortality outcomes. In addition, the study aims to describe the microbiological characteristics, antifungal resistance profiles and therapeutic management of these infections. By identifying existing gaps in regard to predisposing factors and treatment options, this narrative review intends to enhance the currently scarce body of knowledge surrounding this emerging fungal pathogen.

## 2. Materials and Methods

### 2.1. Search Strategy and Inclusion and Exclusion Criteria

This review presents a narrative synthesis of all documented instances of human infections attributed to *Arthrographis* species. The primary objective was to outline the epidemiological profile and mortality rates associated with these infections. Secondary aims included summarizing data on predisposing factors, clinical manifestations, microbiological traits and available treatment options. A literature search was conducted independently by two reviewers (A.Z. and A.G.) in PubMed/Medline and Scopus databases, covering all records up to 25 October 2025. A structured search strategy was employed, using the terms “*Arthrographis*”, combined with (“infection” OR “bloodstream” OR “pneumonia” OR “osteomyelitis” OR “peritonitis” OR “meningitis” OR “endocarditis”). A structured data extraction template was utilized. Study screening was performed in two stages (title/abstract screening followed by full-text assessment), conducted independently by both reviewers. Any discrepancies identified during study selection were resolved through discussion with a senior investigator (P.I.). Eligible studies included original research on humans, such as case reports, case series and cohort studies that provided epidemiological or clinical outcome data related to *Arthrographis* infections. Only articles written in English were considered. Publications such as reviews, systematic reviews, animal studies or papers lacking full-text access or essential epidemiological or mortality data were excluded. To ensure comprehensive coverage, the reference lists of all included studies were additionally screened manually to identify further eligible reports.

### 2.2. Data Extraction and Definition

For every study incorporated into this review, information was extracted regarding the publication year, study design, country of origin and patient demographics, such as age and sex. Additional data included relevant medical history; details regarding the infection’s clinical course, including associated complications or symptom duration and microbiological findings; the identified *Arthrographis* species; antifungal susceptibility patterns; therapeutic management and disease outcomes. The relationship between infection and mortality was recorded according to the interpretations and conclusions provided by the original authors of each study.

## 3. Results

### 3.1. Included Studies’ Characteristics

**Figure 1:**
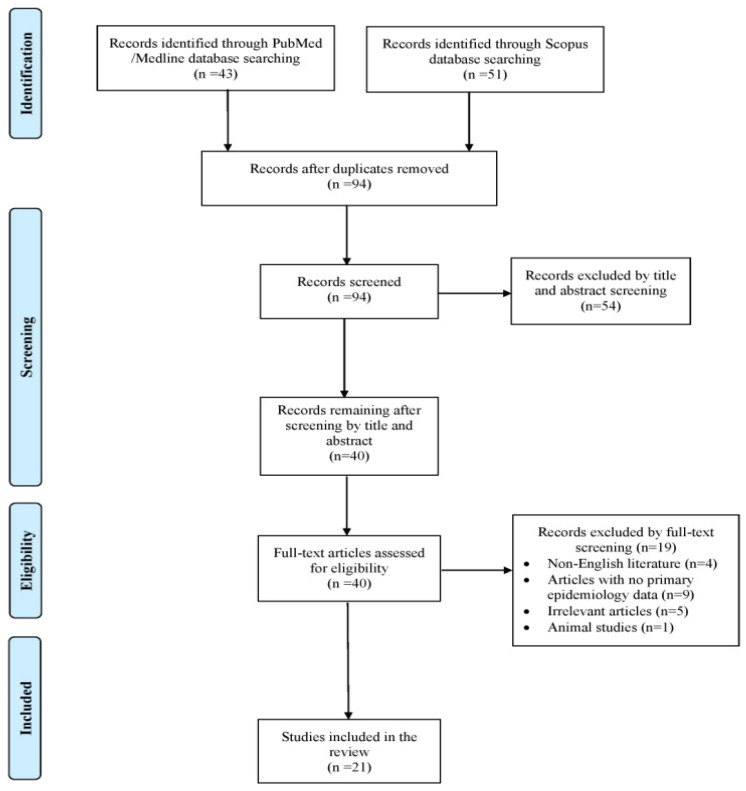
Trial flow of the present review.

A total of 94 articles were initially identified through searches in the PubMed/Medline and Scopus databases. After the removal of duplicates, detailed screenings of the records and the application of the snowball method, only 21 studies met the predefined inclusion criteria and were ultimately included in the final review [2,3,4,5,6,7,8,9,10,11,12,13,14,15,16,17,18,19,20,21,22]. Collectively, these publications described 21 individual patient cases. The study selection process is illustrated in the flowchart presented in Figure 1. Among the reported cases, 11 (52.4%) originated from Europe, 4 (19%) from Asia and America, respectively, and 2 (9.5%) from Australia. All included studies consisted of case reports. Table S1 shows the characteristics of all included studies.

### 3.2. Epidemiology of Arthrographis spp. Infections

The mean age of patients diagnosed with *Arthrographis* spp. infections was 43.62 years, with ages ranging from 7 to 80 years. Of the 21 documented cases, 14 (66.6%) involved male patients. Regarding underlying conditions and predisposing factors, seven individuals (33.3%) had a history of trauma. More specifically, all these cases involved community-acquired trauma; four out of seven cases involved penetrating tissue wounds by steel objects that led to infection, while three cases described foreign body trauma to the eye. Time between trauma and symptom onset ranged between 3 days and 3 years.

A total of five cases (23.8%) had a history of type 2 diabetes mellitus (T2DM) and previous antibiotic administration, respectively. For this review, previous antibiotic administration concerned only the three previous months to symptom onset. Regarding patients with a history of T2DM, ages ranged from 19 to 68 years. Keratitis constituted the most commonly developed infection in three out of five cases; there was also one case of bloodstream infection and one case of pneumonia. Only one death was reported amongst all diabetic patients, while the remaining four successfully recovered. Notably, four patients (19%) were contact lens users, while in another four cases, exposure to soil was described. Concerning contact lens users, all four cases developed keratitis; interestingly, one case also developed concomitant endophthalmitis. All patients successfully recovered with intravenous antifungal treatment, while therapeutic keratoplasty was performed in two out of these four cases. Additionally, immunosuppression, malnutrition and renal failure were present in two patients (9.5%) each. Two individuals (9.5%) had a history of hematological malignancy or lung disease, respectively. Interestingly, both cases of lung disease included cystic fibrosis patients. Only one individual (4.8%) underwent organ transplantation (both lungs) and another one was subjected to surgery (laser eye surgery) within the past three months. A detailed summary of the demographic and clinical characteristics of *Arthrographis* spp. infections’ cases is provided in Table 1.

### 3.3. Clinical Manifestations of Arthrographis spp. Infections

Eyes were the most commonly infected organs, with keratitis appearing in eight patients (38.1%) and conjunctivitis or endophthalmitis in three (14.3%), respectively. In three of these patients, concomitant keratitis and either endophthalmitis or conjunctivitis were observed. Bone and/or joint infection was documented in five patients (23.8%), while three individuals (14.3%) developed *Arthrographis* spp. pneumonia. The central nervous system (CNS), upper respiratory system, skin and bloodstream were infected in two patients each (9.5%). There was only one report (4.8%) of endocarditis and nail infection, respectively.

In 14 cases (66.6%), infection was only locally manifested, while in the remaining 7 cases (33.3%), it was invasive. Regarding patients who developed invasive infection, all demonstrated certain severe risk factors, depicted in Table 2. It is notable that all deaths recorded in the present review were those of patients with invasive infection.

Fever, as well as abscess formation, were the most frequent clinical manifestations of *Arthrographis* infections, observed in six patients (28.6%), respectively, followed by organ dysfunction in four (19%). The CNS (2/4 patients) and the eyes (2/4 patients) were the most commonly affected. Additionally, three patients (14.3%) experienced embolic phenomena; in two of them (66.6%), the emboli affected the CNS, while one individual developed emboli in the liver, left renal artery and left iliac artery. Two patients (9.5%) presented with sepsis and later developed septic shock, while another two (9.5%) exhibited fistula formation or vasculitis, respectively. Moreover, secondary glaucoma and subsequent vision loss were documented in two cases (9.5%). Renal failure was reported in one case (4.8%). Relapse was observed in five individuals (23.8%). The duration of symptoms ranged from acute onset (0 days) to a maximum of 7 months before hospital admission.

### 3.4. Antifungal Resistance and Microbiology of Arthrographis spp. Infections

*Arthrographis* species were identified in corneal scrape cultures in eight patients (38.1%). In contrast, three patients (14.3%) showed growth in respiratory secretions cultures or joint fluid, respectively, while two patients (9.5%) had positive results from pus cultures. The organism was also detected in cultures from blood, cerebrospinal fluid, skin, nail specimen and aortic graft in one case (4.8%), respectively. All isolates were identified as *A. kalrae*, which was the predominant species. No additional *Arthrographis* species were reported. Polymicrobial infections occurred in five patients (23.8%) and involved co-infection with *Aspergillus* spp., *Candida albicans*, *Pseudomonas aeruginosa*, methicillin-resistant *Staphylococcus aureus* (MRSA), *Bacillus cereus* and *Staphylococcus epidermidis.* Pathogen identification was primarily achieved using molecular diagnostic methods. The exact molecular diagnostic applied for identification was reported only in 13 patients (61.9%). Matrix-assisted laser desorption/ionization time-of-flight mass spectrometry (MALDI-TOF MS) successfully confirmed the organism in five cases (38.5%, based on available data). Furthermore, DNA sequencing aided in species identification in another five cases (61.9%), while VITEK was used only in one case (7.7%, based on available data). Antifungal susceptibility testing was conducted in 15 patients (71.4%). Detailed resistance patterns, including the number of patients tested for resistance to specific antifungals and the subsequent resistance percentages, are presented in Table 3. Each isolate underwent testing with a unique combination of antifungal agents. Sensitivity was observed in every isolate tested for voriconazole, itraconazole, miconazole and terbinafine; nine out of nine isolates assessed for voriconazole and nine out of nine for itraconazole resistance were sensitive. Similarly, two out of two isolates tested for miconazole and two out of two tested for terbinafine resistance were also sensitive to these antifungals.

### 3.5. Treatment and Outcome of Arthrographis spp. Infections

Based on the available data, 20 patients (95.2%) received antifungal therapy. Amphotericin B was the most frequently administered agent, prescribed in 11 patients (55%), followed by voriconazole in 8 (40%) and itraconazole in 6 (30%). Fluconazole was administered in five patients (25%), whereas natamycin was used in four patients (20%). Terbinafine and posaconazole were used in three patients (15%) each. Only two patients (10%) received miconazole and one (5%) caspofungin. A combination of antifungal agents was administered to 13 patients (61.9%). Additionally, 13 patients (61.9%) had received antimicrobial agents prior to the definitive diagnosis. Surgical intervention combined with antifungal therapy was undertaken in 13 patients (61.9%). The type of surgical procedure depended on the particular organ infected; for eye infections, it mainly involved corneal debridement, keratoplasty or, in one case, enucleation, while for joint infections it consisted of joint replacement. Among survivors, the median duration of antifungal treatment was 3 months, ranging from one week in a keratitis patient to lifetime treatment in a patient with invasive bone infection.

The overall mortality rate was 19% (4 of 21 patients), with *Arthrographis* infection specifically accounting for 14.3% (3 of 21 deaths). Subgroup analysis revealed mortality rates of 25% (1 of 4) among T2DM and immunosuppressed patients, as well as individuals with recent antimicrobial administration. Moreover, one out of four of the deceased patients had undergone lung transplantation or was suffering from cystic fibrosis, malnutrition or HIV infection, respectively. Mortality was highest among patients who had a history of renal failure; specifically, in two out of four patients. Deceased patients exhibited several risk factors predisposing them to severe disease. The infection site differed across the deceased patients, but all had severe invasive infections. Specifically, one patient had pneumonia and fungemia; another patient exhibited CNS and upper respiratory infection. The third deceased individual had fungemia and endocarditis, while the fourth had CNS infection. Additionally, subgroup analysis revealed a mortality rate of 15.4% (2 out of 13 patients) among patients who underwent surgical procedures; both deaths were closely related to the infection. In one out of four reported deaths, a clear delay in diagnosis and treatment was reported.

## 4. Discussion

Taking into account the existing literature, this is the first narrative review to collate information on the identification and management of *Arthrographis* spp. infections. The present review provides a comprehensive overview of human infections caused by *this rare pathogen*, drawing on data from multiple studies examining epidemiology, microbiology, clinical presentations and therapeutic strategies, as well as disease outcomes. By integrating these findings, this study offers new insights into the pathogen’s epidemiological footprint and its potential clinical impact, which have not been previously examined in a unified manner. Notably, *Arthrographis* spp. differs from more common moulds or yeasts by their slow growth, arthroconidial morphology and potential for misidentification, which may delay diagnosis and treatment. Clinically, the pathogen also displays an unusually broad infection spectrum relative to its rarity, further distinguishing it from more frequently encountered fungal pathogens. This review highlights that infections caused by *Arthrographis* are associated with a relatively high overall mortality rate.

Given the small number of *Arthrographis* spp. infections documented in the current literature, establishing a precise epidemiological profile remains difficult [10]. The diagnosis of this uncommon infection largely depends on the clinician’s level of suspicion, highlighting the need for awareness of its potential occurrence. In the present review, the majority of reported cases involved male patients, with a mean age of 43.62 years. Interestingly, most reported cases originated from Europe, followed by the USA and Asia. Only two cases have been described from Australia. A higher prevalence of the infection in European countries may reflect the difference in exposure to soil, compost or other environmental reservoirs of *Arthrographis* spp. between Europe and the USA or Asia [4]. Differences in fungal taxonomy and naming conventions could mean that some cases of *Arthrographis* spp. in the USA were reported under other genera/species and thus not recognized in a genus-specific review. Notably, differences in surveillance systems, case-reporting practices and publication bias can vary markedly between continents, affecting the apparent geographic distribution of rare infections, while language or cultural practices in scientific publication may also play a role in the incidence of these infections worldwide [23]. Despite existing reports, the paucity of global research prevents the formulation of definitive epidemiological insights into *Arthrographis* infections.

*Arthrographis* spp. constitutes an emerging yeast and environmental saprophyte commonly isolated from soil and compost. The genus comprises five recognized species, including *A. kalrae*, *A. cuboidea*, *A. lignicola*, *A. pinicola* and *A. alba*. Among these species, *A. kalrae* is the most frequently encountered species, exhibiting a global distribution and a natural habitat in soil and decomposing organic matter [1]. Of note, in the present narrative review, it was the only species detected in all documented cases. *A. kalrae*, formerly referred to as *Oidiodendronkalrai*, is an asexual, ascomycetous, filamentous fungus that produces single-celled, hyaline, smooth-walled arthroconidia. These arthroconidia arise either from the fragmentation of undifferentiated hyphae or, in fresh cultures, through disjunction and segmentation of hyaline fertile branches located at the tips of the conidiophores. As they mature, they increase in size and become elongated. Additionally, single-celled, hyaline, smooth, spherical blastoconidia can develop directly on the sides of undifferentiated hyphae or on short pedicels [2]. *A. kalrae* was initially described by Cochet et al. in 1939 [24] in a patient with a nail infection [5]. Generally regarded as an opportunistic pathogen, *A. kalrae* shows a predilection for collagen-rich tissues, such as nails and corneas. Nonetheless, invasive infections have also been observed, including pneumonia, endocarditis, meningitis or sinusitis [4]. Immunosuppression has been identified as a significant risk factor for infection with this fungus, although immunocompetent individuals can also be affected [10].

*Arthrographis* species display notable adaptive versatility, allowing them to cause a wide range of infections that depend on both the route of entry and the immune status of the host. These fungi are often reported as opportunistic pathogens of the respiratory tract in patients with cystic fibrosis [4,13]. Differentiating colonization from true infection is especially challenging in these patients, who frequently exhibit chronic colonization as a result of structural changes in the bronchial tree [17]. When these pathogens enter the bloodstream, they can cause systemic and disseminated infections, often exhibiting neurotropism and affecting deep organs such as the brain, lungs and heart. Although invasive infections are rare, they can also arise during outbreaks linked to direct iatrogenic inoculation. The most commonly reported clinical manifestations in the literature include ocular involvement, especially keratitis, followed by pulmonary infections and joint infections [3,17]. In accordance with this data, our review also identified keratitis as the most common infection site. Previous trauma or insertions of contact lenses or pericardial patches have been recognized as potential sources of infection [13]. This underscores the opportunistic behaviour of the fungus and the need for clinicians to remain vigilant during invasive procedures or when medical devices are in use. Severe infections can also develop in the absence of prior injury, including central nervous system involvement, pulmonary infections and fungemia [4,6,10].

Regarding predisposing risk factors, a history of trauma emerged as the most commonly reported condition among affected patients, as highlighted by this review. In all cases with documented trauma, infections were primarily localized to tissues surrounding the injury, most commonly involving bones and joints at the traumatized site, skin or eyes, with keratitis being the most frequent ocular manifestation [12,22]. Collagen-rich tissues such as nails, cornea or joints are particularly susceptible since *Arthrographis* exhibits a predilection for these structures. Trauma can also trigger necrosis or hematoma formation, further facilitating fungal colonization and proliferation. Moreover, medical interventions, like internal fixation, prosthetic implants or even contact lenses, can serve as surfaces for fungal adherence and biofilm formation, increasing infection risk [9,18]. Previous history of diabetes mellitus (DM) also emerged as a significant risk factor among affected individuals. Although no large studies have been conducted to clarify the role of DM in the development of *Arthrographis* spp. infections, a possible explanation could be that hyperglycaemia negatively affects neutrophil function, including chemotaxis, phagocytosis and intracellular killing of pathogens. It also impairs T-cell-mediated immunity, weakening the host’s defence against opportunistic fungi such as *Arthrographis*, thus increasing susceptibility to both superficial and invasive fungal infections [25,26]. Additionally, elevated glucose levels can act as a nutrient source, supporting fungal growth at sites of colonization or trauma [12]. DM often causes microvascular disease, reducing tissue perfusion and oxygenation; poorly oxygenated tissues provide a favourable environment for fungal colonization and proliferation, especially in skin, nails and soft tissues.

The use of contact lenses as well as soil exposure also appeared to be potential predisposing factors for *Arthrographis* infections. Contact lens usage can cause microscopic abrasions on the corneal epithelium, compromising the eye’s natural barrier. Fungal spores can adhere to contact lenses, storage cases or even contaminated disinfectant solutions. Improper hygiene, infrequent solution changes or use of tap water for cleaning create an environment where *Arthrographis* can survive and later be transferred to the eye [5,27]. Additionally, the space between the contact lens and corneal surface provides a warm, moist, nutrient-rich niche that can support fungal growth. Finally, *A. kalrae* has demonstrated the ability to form biofilms, which can adhere to the lens surface and resist cleaning solutions and host immune defences. Once established, these biofilms make eradication difficult and promote persistent infection [27]. Regarding soil exposure, it often leads to minor skin abrasions, puncture wounds or ocular injuries; such trauma can occur during gardening, agriculture, construction or outdoor work [7,20]. In a case report by Perlman et al., *Arthrographis* keratitis was observed in an individual engaging in gardening work [15]. Fungal spores present in soil or compost can become aerosolized, especially during soil handling or composting. Inhalation of these spores can lead to respiratory tract colonization or infection, particularly in immunocompromised individuals.

Identification of *Arthrographis* species remains difficult, since most clinical microbiology laboratories do not have access to advanced molecular diagnostic methods such as genetic sequencing. The initial step in diagnosing *Arthrographis* spp. infections generally involves microscopic examination; microbiological differentiation of *Arthrographis* is often challenging. Morphologically, its colonies range from creamy white to tan and exhibit slow to moderate growth. Initially, the organism appears yeast-like before developing hyphal structures. This growth pattern can lead to it being mistaken for other fungi, such as *Chrysoniliasitophila* and *Candida albicans* [11,14]. Accurate identification of fungal species in clinical mycology increasingly relies on molecular methods, including MALDI-TOF MS and sequencing of the internal transcribed spacer (ITS) regions of ribosomal DNA. Among these, MALDI-TOF MS and DNA sequencing emerged in the present review as the techniques most frequently used for identifying *Arthrographis* species. In general, MALDI-TOF MS represents an effective diagnostic approach for achieving rapid identification of this uncommon pathogen. Complementary techniques, including PCR, may also support the diagnosis of *A. kalrae* [11,18]. In a case report by Xi et al., for identification purposes, the D1–D2 domains and ITS regions of ribosomal DNA were sequenced from both the isolate and the *A. kalrae* UAMH 3616-type strain. Sequence comparison revealed complete (100%) homology across the D1–D2 (604 bp) and ITS (508 bp) regions [18]. Due to the high cost and restricted accessibility of advanced molecular techniques, precise identification should integrate biochemical and cultural features characteristic of the pathogen. In all included cases, biological specimen cultures were performed, yielding both biochemical and cultural findings. A positive fungal culture may therefore constitute the initial laboratory clue, warranting further confirmation through advanced molecular diagnostic tools.

To date, standardized antifungal susceptibility breakpoints for *Arthrographis* species have not been defined, due to the limited number of comprehensive investigations into their susceptibility characteristics [4]. As a result, the antifungal susceptibility findings summarized in this review are predominantly based on individual case reports. A recent study by Sandoval-Denis et al. evaluated the antifungal susceptibility of 22 *A. kalrae* strains [28]. The azoles demonstrated strong activity (mean MIC = 0.46 µg/mL), whereas amphotericin B exhibited limited efficacy (mean MIC = 2 µg/mL) and, notably, all echinocandins showed no in vitro activity (mean MIC at 24 h > 8 µg/mL). These findings were consistent with those reported by the National Reference Centre for Invasive Mycoses and Antifungals, which employs the EUCAST methodology [28]. The isolates included in this review were largely susceptible to voriconazole, itraconazole, miconazole and terbinafine, but displayed increased resistance to fluconazole, flucytosine and echinocandins, with caspofungin showing complete resistance (100%). The mechanisms underlying antifungal resistance in these pathogens remain poorly understood, highlighting the need for further investigation. Equally important is the establishment of standardized interpretive criteria for susceptibility testing to provide accurate and reproducible assessments of antifungal efficacy.

Managing *Arthrographis* spp. infections is difficult since standardized antifungal treatment guidelines are lacking. Prompt and intensive therapy is advised in systemic cases to avert severe or fatal outcomes [4,20]. Treatment typically involves both antifungal agents and surgical procedures [17]. Antifungal susceptibility testing is recommended to guide treatment decisions, with preference given to agents demonstrating the lowest MICs and favourable pharmacokinetic properties for effective penetration at the site of infection. In in vitro studies and case reports, terbinafine has emerged as one of the most active agents and has been used successfully in onychomycosis [7]. Among azoles, posaconazole, itraconazole or voriconazole have shown efficiency and have been used in reported cases of bone, joint or soft tissue infection [3]. Amphotericin B appears to have only moderate to limited activity, and echinocandins appear to have minimal or no in vitro activity against *A. kalrae*. Clinically, successful management includes combinations such as liposomal amphotericin B and posaconazole or itraconazole in deep soft tissue infections, often for extended durations to achieve eradication [14,18]. The length of antifungal therapy depends on the severity of clinical presentation. In this review, treatment durations ranged from 7 days to 3 years or more prolonged in long-term survivors, especially in patients with serious invasive infections [3,20]. In 13 reported cases, surgery was undertaken alongside antifungal treatment; the type of surgical procedure depended on the site of infection. In cases of keratitis, keratoplasty was commonly applied, while in bone infections, surgical debridement was conducted [3,8].

Infections provoked by *A. kalrae* are frequently associated with severe systemic illness, with reported mortality rates reaching nearly 19.04% in the present study. These infections are linked to relatively high mortality, particularly among patients with significant underlying conditions or those who developed complications such as multiple organ failure [4,6]. This high fatality rate is often attributed to delayed or incorrect diagnosis, commonly resulting from misidentification of the pathogen as another fungal or bacterial species. Additionally, the organism’s ability to induce aggressive infections, coupled with limited diagnostic availability and incomplete knowledge of its resistance mechanisms, likely contributes to the substantial mortality observed.

This study has certain limitations. Firstly, the literature search may not have identified all relevant studies on epidemiology and mortality, since some publications could have been missed due to the search strategy. Secondly, the analysis relied solely on case reports and case series, which are inherently dependent on the accuracy of the documented data. Thirdly, many studies did not use molecular methods such as genetic sequencing, raising the possibility of misidentification. In addition, incomplete reporting in some cases limited the comprehensiveness of the analysis. Finally, restricting the review to English-language articles may have introduced selection bias, although the number of studies excluded for this reason was minimal.

## 5. Conclusions

This narrative review provides the first comprehensive synthesis of epidemiological, clinical and microbiological characteristics; antifungal susceptibility patterns; treatment strategies and clinical outcomes associated with infections provoked by *Arthrographis* species, offering novel insights that were previously unavailable in the literature. Special attention is given to highlighting the pathogenic capacity of this relatively understudied genus. Among the species identified, *A. kalrae* was the only one isolated, with eyes being the most frequently infected organ. The pathogen exhibited resistance to various antifungal agents. Even though formal treatment guidelines are lacking, amphotericin B and voriconazole were the most commonly used antifungals. Prompt initiation of antifungal therapy, preferably guided by in vitro susceptibility testing, remains critical. Due to the opportunistic nature of *Arthrographis* spp. and the diagnostic challenges posed by limitations in commercial identification systems, increased awareness among clinicians and microbiologists is crucial for timely recognition and effective management. Despite certain limitations, this review highlights the need for future longitudinal and controlled studies to further understand *Arthrographis* infections and to establish evidence-based treatment strategies.

## Figures and Tables

**Table 1:** Characteristics of patients with *Arthrographis* species infections.

Characteristic	All Patients (*n* = 21)	Survived (*n* = 17)	Died (*n* = 4)
Age, years, mean (SD)	43.62 (24.19–63.05)	45.59 (25.12–66.06)	35.25 (22.33–48.17)
Male gender, *n* (%)	14 (66.6)	12 (70.6)	2
Predisposing factors			
Trauma, *n* (%)	7(33.3)	7 (41.2)	0
T2DM, *n* (%)	5(23.8)	4 (23.5)	1
Previous antibiotics *, *n* (%)	5 (23.8)	4 (23.5)	1
Contact lenses, *n* (%)	4 (19)	4 (23.5)	0
Soil exposure, *n* (%)	4 (19)	4 (23.5)	0
Immunosuppression, *n* (%)	2 (9.5)	1 (5.9)	1
Polymicrobial infection, *n* (%)	5 (23.8)	4 (23.5)	1
Clinical characteristics			
Fever, *n* (%)	6 (28.6)	4 (23.5)	2
Abscess, *n* (%)	6 (28.6)	6 (35.3)	0
Treatment			
Amphotericin B, *n* (%)	11 (52.4	10 (58.8)	1
Voriconazole, *n* (%)	8 (38.1)	7 (41.2)	1
Itraconazole, *n* (%)	6 (28.6)	5 (29.4)	1
Fluconazole, *n* (%)	5 (23.8)	5 (29.4)	0
Natamycin, *n* (%)	4 (19)	4 (23.5)	0
Outcomes			
Deaths due to infection, *n* (%)	3 (14.3)	NA	NA
Deaths overall, *n* (%)	4 (19)	NA	NA

T2DM: type 2 diabetes mellitus, NA: not applicable, *: within the past 3 months.

**Table 2:** Characteristics of patients with invasive *Arthrographis* spp. Infections.

Author/Reference/Year	Age/Sex	Type of Invasive Infection	Risk Factors	Outcome
Denis et al., 2016 [4]	19/F	Lower Respiratory	T2DM, CF, RF, OT, Malnutrition	Deceased
Chin-Hong et al., 2001 [6]	33/M	CNS, Upper Respiratory	ISx, RF, HIV, previously on antibiotics *, CMV retinitis with left-eye blindness, *Pneumocystis carinii* pneumonia, disseminated *Mycobacterium avium* infection, oral candidiasis	Deceased
Vos et al., 2012 [10]	61/M	Lower Respiratory	History of hematologic malignancy, wedge resection and pleurectomy due to recurrent right-sided pneumothorax	Survived
De Diego Candela et al., 2010 [13]	50/F	Endocarditis, Fungemia	Metallic cardiac valve	Deceased
Fiscarelli et al., 2019 [16]	7/M	Lower Respiratory	CF, previously on antibiotics *	Survived
Xi et al., 2004 [18]	39/M	Bones/Joints, Upper Respiratory, Endophthalmitis	Trauma, Soil exposure	Survived
Pichon et al., 2008 [21]	39/M	CNS	Malnutrition, cattle breeding, tobacco/alcohol abuse	Deceased

M: male; F: female; ISx: immunosuppression; T2DM: type 2 diabetes mellitus; CNS: central nervous system; CMV: cytomegalovirus; CF: cystic fibrosis; OT: organ transplantation; HIV: human immunodeficiency virus; RF: renal failure; *: antibiotic administration within the past 3 months.

**Table 3:** Antifungal resistance patterns.

Antifungal Agent	Number of Patients	Resistance (%)
Caspofungin	3/3	100
Anidulafungin	1/1	100
Flucytosine	5/6	83.3
Fluconazole	6/8	75
Micafungin	3/4	75
Natamycin	1/3	33.3
Posaconazole	1/6	16.6
Amphotericin B	1/14	7.1

## Data Availability

No new data were created or analyzed in this study.

## Supplementary Materials

The following supporting information can be downloaded at https://www.mdpi.com/article/10.3390/pathogens15010112/s1: Table S1: Characteristics of all included studies [2,3,4,5,6,7,8,9,10,11,12,13,14,15,16,17,18,19,20,21,22].
