# Supplementary material for: Arthrographis Infections in Humans—A Narrative Review
Source: Pathogens. 2026 Jan 20;15(1):112. doi: 10.3390/pathogens15010112 (PMC12845145; doi:10.3390/pathogens15010112)
Supplement: Supplementary file 1 [file pathogens-15-00112-s001.zip › Table S1.pdf]

Supplementary Materials

Table S1. Characteristics of all included studies.

Author name/Reference Number	Year	Age (years)	Gender	Comorbidities	Type of Infection	Antifungal Treatment	Surgery	Fatality
Denis et al. [4]	2016	19	F	T2DM, CF, RF, OT, Malnutrition	Lower Respiratory	Amphotericin B, Voriconazole, Caspofungin	-	+
Chin-Hong et al. [6]	2001	33	M	ISx, RF, HIV, previously on antibiotics*, CMV retinitis with left-eye blindness, *Pneumocystis carinii* pneumonia, disseminated *Mycobacterium avium* infection, oral candidiasis	CNS, Upper Respiratory	Itraconazole	+	+
Sugiura et al. [7]	2010	63	M	-	Nail	Miconazole, Terbinafin	-	-
Huang et al. [8]	2024	61	M	T2DM, Contact lenses	Keratitis/ Endopthalmitis/ Conjuc-tivitis	Amphotericin B, Voriconazole, Natamycin	+	-
Sainaghi et al. [9]	2015	68	M	T2DM, Trauma	Bones/Joints	Voriconazole	+	-
Vos et al. [10]	2012	61	M	History of hematologic malignancy, wedge resection and pleurectomy due to recurrent right-sided pneumothorax	Lower Respiratory	Itraconazole	+	-
Thomas et al. [11]	2011	42	F	Contact lenses	Keratitis	Voriconazole	+	-
Boan et al. [3]	2012	33	M	Trauma	Bones/ Joints	Amphotericin B, Voriconazole, Fluconazole, Posaconazole, Terbinafin	+	-
Ting et al. [5]	2020	65	M	Active hematologic malignancy, Isx, Chemotherapy, prior HSV-related keratouveitis	Keratitis/ Endopthalmitis/ Con-junctivitis	Amphotericin B, Voriconazole, Fluconazole, Natamycin	+	-
Ramli et al. [12]	2012	52	M	T2DM, Trauma	Keratitis	Amphotericin B, Fluconazole	-	-
De Diego Candela et al. [13]	2010	50	F	Metallic cardiac valve	Endocarditis, Fungemia	Posaconazole	+	+
Biser et al. [14]	2004	23	F	Contact lenses, Soil exposure, Previously on antibiotics*	Keratitis/ Conjunctivitis	Amphotericin B, Itraconazole, Miconazole	-	-
Perlman et al. [15]	1997	49	F	Contact lenses, Soil exposure	Keratitis	Amphotericin B, Natamycin, Ketokonazole	-	-
Fiscarelli et al. [16]	2019	7	M	CF, Previously on antibiotics*	Lower Respiratory	Itraconazole	-	-
Fernández-Barrientos et al. [2]	2020	38	F	Recent laser surgery**, Previously on antibiotics*	Keratitis	Voriconazole, Natamycin	+	-
Lemaigre et al. [17]	2025	9	NR	Trauma, Soil exposure	Bones/ Joints	Amphotericin B, Voriconazole	+	-
Xi et al. [18]	2004	39	M	Trauma, Soil exposure	Bones/ Joints, Upper Respiratory, Endopthalmitis	Amphotericin B, Fluconazole, Itraconazole, Nystatin	+	-
Degavre et al. [19]	1997	80	M	Previously on antibiotics*	Skin	Itraconazole	-	-
Ong et al. [20]	2014	33	M	Trauma	Skin, Bones/ Joints	Amphotericin B, Posaconazole, Terbinafin	+	-
Pichon et al. [21]	2008	39	M	Malnutrition, Cattle breeding, Tobacco/ Alcohol abuse	CNS	-	-	+
Chow et al. [22]	2014	52	M	T2DM, Trauma	Keratitis	Amphotericin B, Fluconazole	+	-

NR: Not Reported, M: Male, F: Female, ISx: Immunosuppression, T2DM: Type 2 Diabetes Mellitus, CNS: Central Nervous System, CMV: Cytomegalovirus, CF: Cystic Fibrosis, OT: Organ Transplantation, HIV: Human Immunodeficiency Virus, RF: Renal Failure, *: antibiotic administration within the past 3 months, **: laser surgery within the past 4 months.
